# Differential Expression Profiles in the Midgut of *Triatoma infestans* Infected with *Trypanosoma cruzi*

**DOI:** 10.1371/journal.pone.0061203

**Published:** 2013-05-02

**Authors:** Diego S. Buarque, Glória R. C. Braz, Rafael M. Martins, Anita M. Tanaka-Azevedo, Cícera M. Gomes, Felipe A. A. Oliveira, Sergio Schenkman, Aparecida S. Tanaka

**Affiliations:** 1 Department of Biochemistry, Escola Paulista de Medicina, Universidade Federal de São Paulo, São Paulo, Brazil; 2 Department of Biochemistry, Instituto de Química, Universidade Federal do Rio de Janeiro, Rio de Janeiro, Brazil; 3 Biology of Host Parasite Interactions Unit, Institut Pasteur, Paris, France; 4 Laboratory of Herpetology, Instituto Butantan, São Paulo, Brazil; 5 Department of Microbiology, Immunology and Parasitology, Universidade Federal de São Paulo, São Paulo, Brazil; Albert Einstein College of Medicine, United States of America

## Abstract

Chagas disease, or American trypanosomiasis, is a parasitic disease caused by the protozoan *Trypanosoma cruzi* and is transmitted by insects from the Triatominae subfamily. To identify components involved in the protozoan-vector relationship, we constructed and analyzed cDNA libraries from RNA isolated from the midguts of uninfected and *T. cruzi*-infected *Triatoma infestans*, which are major vectors of Chagas disease. We generated approximately 440 high-quality Expressed Sequence Tags (ESTs) from each *T. infestans* midgut cDNA library. The sequences were grouped in 380 clusters, representing an average length of 664.78 base pairs (bp). Many clusters were not classified functionally, representing unknown transcripts. Several transcripts involved in different processes (e.g., detoxification) showed differential expression in response to *T. cruzi* infection. Lysozyme, cathepsin D, a nitrophorin-like protein and a putative 14 kDa protein were significantly upregulated upon infection, whereas thioredoxin reductase was downregulated. In addition, we identified several transcripts related to metabolic processes or immunity with unchanged expressions, including infestin, lipocalins and defensins. We also detected ESTs encoding juvenile hormone binding protein (JHBP), which seems to be involved in insect development and could be a target in control strategies for the vector. This work demonstrates differential gene expression upon *T. cruzi* infection in the midgut of *T. infestans*. These data expand the current knowledge regarding vector-parasite interactions for Chagas disease.

## Introduction

Parasitic diseases transmitted by arthropods have been some of the most severe causes of human death in the world, especially in developing countries. Malaria, yellow fever, dengue and African trypanosomiasis are examples of arthropod-borne diseases transmitted to humans [1]; Chagas disease, or American trypanosomiasis, is another example of this type of disease. Chagas disease is caused by the protozoan parasite *Trypanosoma cruzi* and is transmitted by insects from the Triatominae subfamily [2].

Chagas disease remains prevalent in many Latin American countries, affecting an estimated eight million people [3], and it is correlated with poor living conditions. Although most acute infections are asymptomatic, approximately 30% become chronic, resulting in approximately 12,500 deaths annually. Moreover, there is neither a vaccine nor a preventive treatment to cure Chagas disease, as the drugs currently used have efficacies only in the acute phase of the disease, leading to several side effects in humans [3], [4]. In contrast to the majority of parasites that transmit arthropod-borne diseases, *T. cruzi* is not inoculated in the host's saliva because this protozoan does not infect the salivary glands of the vector insect. Instead, the parasite colonizes the intestinal tract and rectum of triatomines. As part of the feeding process, the insect defecates, and its feces, containing *T. cruzi*, remain on the skin of the vertebrate. Then, the host becomes infected via mucosa or at bite sites [5].

*T. cruzi* uses a blood feeding process to proliferate and develop inside the insect's midgut. However, the parasite must bypass the vector's defenses, which are composed of innate immunity molecules expressed in response to different types of infection [6]. The midgut of blood-sucking triatomines is considered an immune competent tissue [4], and it is suggested that inducible immune compounds from the intestinal tract can modulate parasite development [7]–[9]. Nevertheless, little information has been published regarding expression profiles in the midgut or the role of innate immunity for many important disease vectors [10], including triatomine bugs such as *Triatoma infestans*, which is one of the most important vectors of *T. cruzi* [11].

Studies on the molecular interactions of protozoa from the *Trypanosoma* genus and triatomine vectors are limited. Most of these studies address the interactions of parasites in the hemolymph as they move to the salivary glands of insects. In the case of *T. cruzi*, this parasite remains in the intestinal tract and therefore has minimal or no direct contact with hemolymph factors [12]. Thus, we aimed to identify molecules from the vector midgut that could be involved in the parasite-vector relationships using Expressed Sequence Tags (ESTs) sequencing and analysis.

The study of genomes from disease vectors is a helpful technique to identify targets with the aim of controlling insect development and parasite transmission. Although the genome sequencing of the triatomine insect *Rhodnius prolixus* is in progress, there are currently there no efforts to sequence the *T. infestans* genome [11].

EST analysis is an alternative to genome studies that helps provide information about disease vectors. Some approaches that have been used to identify Chagas disease vector molecules include the analysis of expressed sequence tags (ESTs) from the salivary glands [13]–[15] and ovaries [16] of triatomine insects. However, no extensive investigations have been conducted concerning the *T. infestans* midgut.

In this work, we analyzed midgut cDNA sequences from insects infected with *T. cruzi* in comparison to uninfected *T. infestans*. Several ESTs matched with putative proteins related to the protection of triatomine insects against parasite challenges, which were modulated in infected *T. infestans* midguts. Expression patterns of some of these molecules were confirmed by qRT-PCR. This is the first EST profile analysis in the midgut of a triatomine insect infected with *T. cruzi*, which provides new insights towards understanding the role of midgut molecules in triatomine-*T. cruzi* interactions.

## Results and Discussion

### *T. infestans* EST sequencing profile

Expressed Sequencing Tag profiling of insects under stress or parasitic infection could provide information about related cellular functions, including growth, development and immune defense [17]. A total of 1,341 clones (661 and 680 clones from uninfected and infected insects, respectively) were sequenced to obtain ESTs from *T. infestans* midgut libraries. These raw data were subjected to cleaning steps, resulting in approximately 440 high-quality ESTs from each library. To obtain clusters, reads of the two libraries were assembled using the CAP3 program, which generated 380 clusters using sequences representing a minimum size of 76 base pairs (bp) (Table 1 and Data S1 – spreadsheet stats). A consensus sequence derived from two or more sequences was named ‘contig,’ and the term ‘singleton’ was used for single sequences. In this section, we will use the denomination ‘cluster’ to address ESTs from both contigs and singletons.

**Table 1:** Analysis summary of *T. infestans* ESTs.

Description	Number	%
High quality ESTs	872	100
Contigs	380	-
Mean (bp)	664.78	-
BLATX NR protein database (<10^−5^)	472	54.12

**Figure 1:**
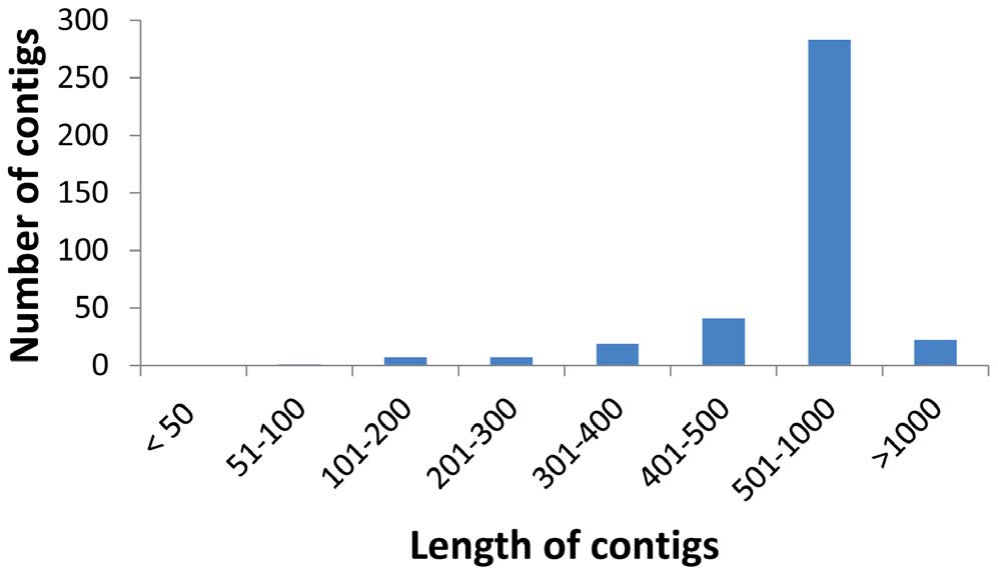
Length distribution of *T. infestans* clusters.

The average cluster length among all ESTs was 664.78 bp, and approximately 283 clusters were distributed in a range of 500–700 bp (Figure 1 and Data S1, spreadsheet stats). The fact that the majority of clusters were over 500 bp in length was an advantage because the production of larger clusters facilitated subsequent functional analysis [18]. Only clusters longer than 200 bp were selected for further analysis.

For functional analysis, ESTs were matched against a subset of the non-redundant (nr) NCBI protein database (see NR-light in methods) using BLASTx. Approximately 55% of whole *T. infestans* midgut ESTs had a match in this database when implementing a cut-off E-value of 1×10^−5^ (Table 1 and Data S1). In the absence of *T. infestans* genomic information, we compared the clusters obtained to a preliminary set of *Rhodnius prolixus* proteins predicted from the genome of this insect. The fact that 241 out of 380 *T. infestans* clusters matched to predicted *Rhodnius* proteins with an E-value lower than 10^−5^ showed that both of these triatomines have similar protein sets. In addition, some differences between these two Chagas disease vectors were observed: 139 of the predicted proteins appeared to be specific to *T. infestans*. From the 241 predicted proteins that matched the *Rhodnius* proteins, we highlighted 33 proteins classified as unknown; these are listed in the worksheet named “*Rhodnius* matches” (Data S1). Most of these sequences presented differential expression upon infection, and their relevance in the efficiency of *T. cruzi* infection will be investigated in future studies.

**Figure 2:**
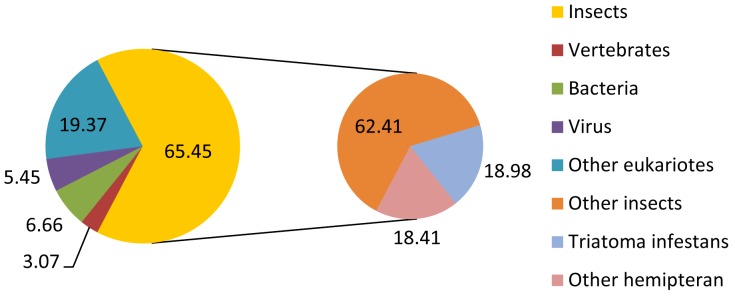
A pie chart showing the species distribution of BLASTx hits of the *T. infestans* clusters for several organisms.

The EST profile was also analyzed in terms of a taxonomic classification by comparison to other taxa (Figure 2). As the computational time to blast sequences to the non-redundant protein database (nr) increased considerably, we built a protein database, named NR-light, that is a subset of nr, as described in the methods section. This database includes many arthropods, viruses, bacteria and protozoan parasites with well annotated genomes that commonly infect arthropods and some vertebrates. Figure 2 presents complementary information about the similarity of *T. infestans* clusters to the annotated proteins when they were blasted against the two datasets.

Because the NR-light database may lead to a bias towards insects, we observed that the highest percentage of clusters matched insects (65.45%), and approximately 19% of the clusters present in this group were similar to the predicted proteins from *T. infestans*. Another abundant class of clusters was related to other Hemipterans (approximately 19%), which corresponded to hits from insects such as *Triatoma dimidiata*, *Triatoma matogrossenssis* and the pea aphid *Acyrthosiphum pisum*, the representative organism of this group (see Data S1). The high number matches to *A. pisum* sequences may be explained by the prevalence of this insect genome [19]. However, triatomine midgut sequences are scarce; therefore, our ESTs enhance the data related to Chagas disease vectors.

We also used a detailed functional classification system (Table 2; Data S1, worksheet class distribution and Data S2, worksheet <5%X column O) and the statistics of cluster classes for upregulated clusters (Table 3; Data S1, worksheet class distribution and Data S2, worksheet up-reg) and downregulated clusters (Table 4; Data S1, worksheet class distribution and Data S2, worksheet down-reg). Many transcripts were classified as putative secreted proteins related to processes such as protein transcription and synthesis.

**Table 2:** Functional classification of transcripts from all clusters.

Class	Number of Clusters	Number of ESTs
Putative secreted proteins	14	62
Nuclear regulation	5	8
Transcription factor	3	6
Transcription machinery	9	25
Protein synthesis machinery	25	42
Protein export machinery	9	12
Protein modification machinery	19	27
Proteasome machinery	2	2
Transporters/storage	10	22
Oxidant metabolism/detoxification	6	23
Metabolism, carbohydrate	6	9
Metabolism, nucleotide	1	1
Metabolism, amino acid	2	2
Metabolism, lipid	10	15
Signal transduction	18	33
Extracellular matrix/cell adhesion	7	17
Cytoskeletal	7	18
Transposable element	3	3
Metabolism, energy	18	50
Unknown	126	288
Unknown, conserved	54	104
Immunity	7	12
Viral	12	73
Nuclear export	1	1
Secreted proteinase inhibitor	6	14

**Table 3:** Functional classification of transcripts that originated from upregulated contigs.

Class	Number of Clusters	Number of ESTs
Putative secreted proteins	3	14
Nuclear regulation	1	2
Transcription factor	1	2
Transcription machinery	2	4
Protein synthesis machinery	4	10
Transporters/storage	4	9
Oxidant metabolism/detoxification	2	9
Metabolism, carbohydrate	2	4
Metabolism, lipid	1	3
Signal transduction	2	7
Extracellular matrix/cell adhesion	1	2
Cytoskeletal	2	6
Metabolism, energy	1	2
Unknown	7	8
Unknown, conserved	4	13
Immunity	1	2
Secreted proteinase inhibitor	1	2

**Table 4:** Functional classification of transcripts that originated from downregulated contigs.

Class	Number of Clusters	Number of ESTs
Putative secreted proteins	4	16
Nuclear regulation	1	3
Transcription machinery	1	11
Protein synthesis machinery	4	11
Protein export machinery	2	4
Protein modification machinery	1	2
Transporters/storage	1	2
Oxidant metabolism/detoxification	1	7
Metabolism, carbohydrate	1	2
Signal transduction	3	20
Extracellular matrix/cell adhesion	2	4
Metabolism, energy	5	15
Unknown	9	33
Unknown, conserved	7	19

A large portion of transcripts comprised identified proteins representing unknown functions (Table 2). A number of these transcripts were found to be upregulated or downregulated in response to *T. cruzi* infection. Specifically, 21 unknown or unknown conserved transcripts were upregulated (Table 3) whereas 52 were downregulated (Table 4) in *T. infestans* upon infection. It is possible that some proteins encoded by these transcripts may have novel functions in response to *T. cruzi* infection and are good targets for future studies.

Differential transcript expression was observed for genes that participate in many processes, such as energy metabolism, detoxification, immunity and proteinase control. Regarding the expression of important molecules involved in these processes and in vector-parasite relationships, we selected some representative contigs among those that were differentially expressed in uninfected insects and in insects infected with *T. cruzi* to quantify expression using qRT-PCR.

### Transcripts differentially expressed in *T. infestans* midgut

#### Down-regulated transcript

**Figure 3:**
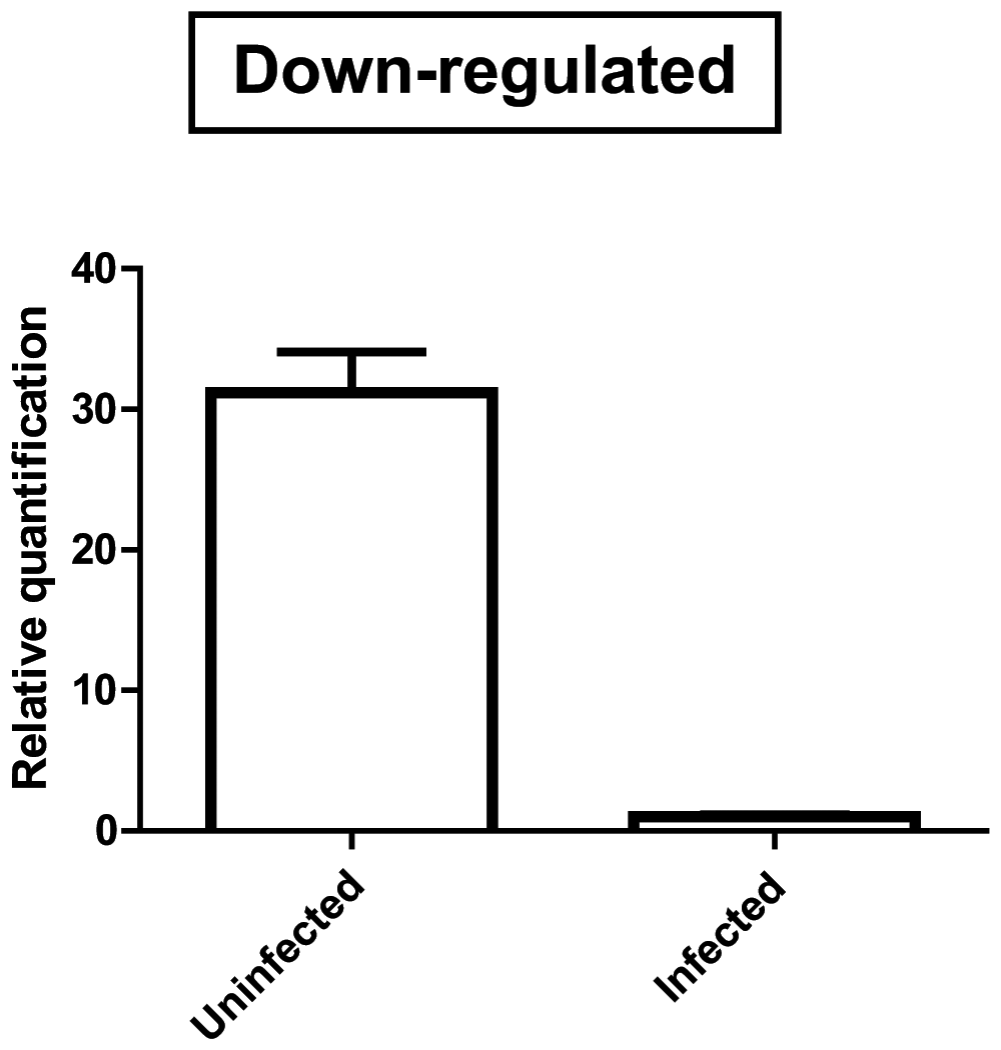
qRT-PCR of Thioredoxin reductase upon *T. cruzi* infection. The levels of mRNA from Thioredoxin reductase were obtained by relative quantification. Adult insects infected with *T. cruzi* and uninfected *T. infestans* were used for analysis (three biological samples were used for both the uninfected and infected groups). All data were normalized to 18S ribosomal RNA, representing the mean (n = 3) of identical triplicates ± standard deviation. An unpaired *t* test was performed for statistical analysis, and differences were considered significant at P<0.05. Asterisks represent significant differences (*** P<0.001).

A contig encoding a Thioredoxin reductase (Data S1, contig 573) was downregulated in infected insects (Figure 3). Thioredoxin reductase is an antioxidant enzyme that promotes the conversion of oxidized thioredoxin and can act together with the glutathione system to regenerate reduced glutathione, contributing to the detoxification of free radicals and impairing oxidative stress in hematophagous insects [20]. According to Paes et al. [21], low concentrations of molecules involved in oxidative stress promote the proliferation of *T. cruzi*. Therefore, the expression of thioredoxin reductase in infected bugs may be modulated by *T. cruzi* to allow parasite proliferation inside the *T. infestans* midgut.

#### Up-regulated transcripts

**Figure 4:**
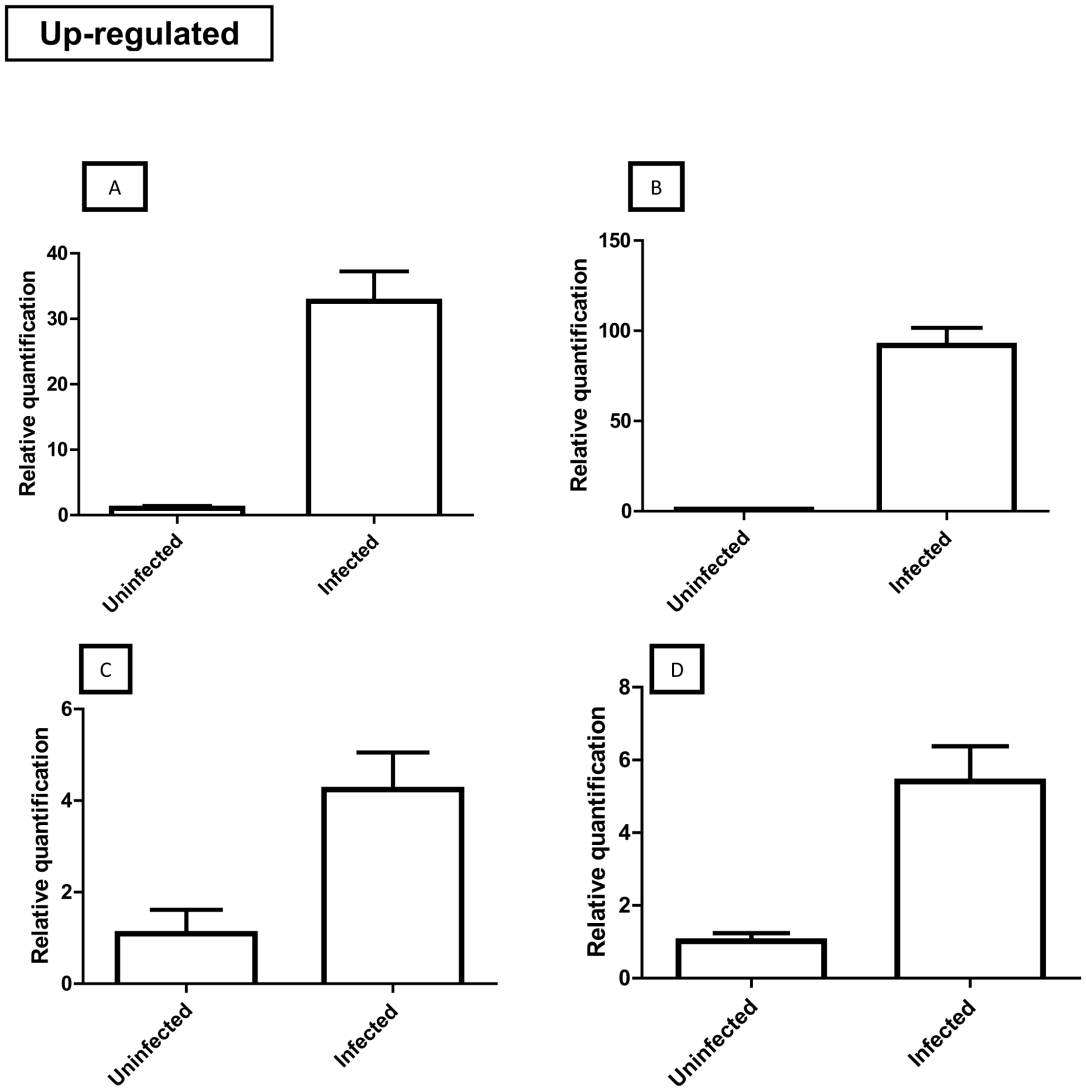
qRT-PCR of four transcripts upregulated upon *T. cruzi* infection. Amounts of mRNA from nitrophorin-like protein (A), 14 kDa protein (B), lysozyme (C) and cathepsin D (D) were obtained by relative quantification. Adult insects infected with *T. cruzi* and uninfected *T. infestans* were used for analysis (three biological samples were used for both the uninfected and infected groups). All data were normalized to 18S ribosomal RNA, representing the mean (n = 3) of identical triplicates ± standard deviation. An unpaired *t* test was performed for statistical analysis, and differences were considered significant at P<0.05. Asterisks represent significant differences (** P<0.01; *** P<0.001).

Contig 564 (Data S1) was upregulated (approximately 35-fold) in infected *T. infestans* compared to the control (Figure 4A). This contig matched the rhodnius biogenic amine binding-like protein [*Triatoma matogrossensis*] in the NR-light database (Data S1, column U) and presented the best match to nitrophorin in Conserved Domain Database (CDD) (Data S1, column DC). Nitrophorins are nitric oxide carriers that have been reported to play a role in the innate immunity of insects. Although nitrophorins are salivary gland molecules, they are lipocalins and are able to bind non-polar molecules such as nitric oxide [22]. According to Whitten et al. [10], nitric oxide production was upregulated when the triatomine insect *Rhodnius prolixus* was infected with *T. cruzi*. Because nitric oxide can react with other radicals to generate compounds toxic to *T. cruzi* [23], nitrophorin-like molecules might be upregulated through signaling mechanisms carried out by the parasite to aid its survival in the insect's midgut.

We found increased expression (approximately 90-fold) of contig 538 (Data S1) encoding a possible 14 kDa putative secreted protein in infected insects (Figure 4B). These transcripts did not match known proteins in the databases, including the Rhodnius database, suggesting that this is a new protein that has discovered in the midgut of *T. infestans*. Thus, elucidation of the role of this hypothetical protein merits further investigation as it was highly modulated by *T. cruzi* infection and seems to be involved in vector-parasite relationships.

The expression of lysozymes was also upregulated in infected insects (Figure 4C). Lysozymes catalyze the hydrolysis of glycosidic bonds of peptidoglycans present in bacterial cell walls and causes bacterial lysis [24]. Lysozymes are considered to be an antimicrobial peptide expressed in response to bacterial challenges [8]. However, high expression levels of lysozymes were observed upon the artificial injection of *T. cruzi* in the hemolymph of *Rhodnius prolixus* [25], indicating that this protein may also be involved in the modulation of *T. cruzi* infection in triatomine insects.

A contig encoding cathepsin D (Data S1, contig 183) was upregulated in the *T. infestans* midgut when infected with *T. cruzi* (Figure 4D). Cathepsin D is a lysosomal protease involved in digestion processes in triatomine insects. The expression of transcripts encoding cathepsin D was detected in the anterior midgut of *T. infestans* [26]. Borges et al. [27] showed that cathepsin D activity increased when the triatomine insect was infected with *T. cruzi* and that this activity was due to parasite colonization in the midgut.

Cystatin was another molecule that was found to be upregulated upon *T. cruzi* infection. Cystatins are reversible and tight-binding inhibitors of papain-like cysteine proteases, and they are widespread in plants, animals and microorganisms [28]. Although the number of cystatin transcripts remained constant in both libraries, qRT-PCR revealed that this inhibitor is upregulated in the anterior midgut when *T. infestans* is infected with *T. cruzi* (data published in Buarque et al. [29]). Our studies on *T. infestans* cystatins show that recombinant *T. infestans* cystatin (Tigutcystatin) is a tight-binding inhibitor (K*_i_* = 3.29 nM) of the *T. cruzi* cysteine protease cruzipain [29]. Thus, *T. infestans* cystatins might be important in modulating *T. cruzi* colonization inside the insect midgut by inhibiting cruzipain, which is a virulence factor for the parasite *T. cruzi* [30].

In both libraries, we also identified contigs assembled from ESTs with unchanged expression related to several metabolic processes and important for *T. infestans* development. We list some of these contigs below (Table 5).

**Table 5:** A list of contigs with unchanged expression that are important for metabolic processes or defense in *T. infestans*.

Putative protein	Nucleotide length (bp)	E-value	Identity (%)	Species of best match
Infestin	634	1.00E-121	98	*Triatoma infestans*
FABL	624	7.00E-44	77	*Cimex lectularius*
Triabin	779	5.00E-14	31	*Triatoma matogrossensis*
Defensin	525	2.00E-47	92	*Triatoma brasiliensis*
JHBP	915	2.00E-05	22	*Daphnia pulex*

FABL – Fatty acid binding lipocalin.

JHBP – Juvenile hormone binding protein.

### Transcripts with unchanged expression upon *T. cruzi* infection

**Figure 5:**
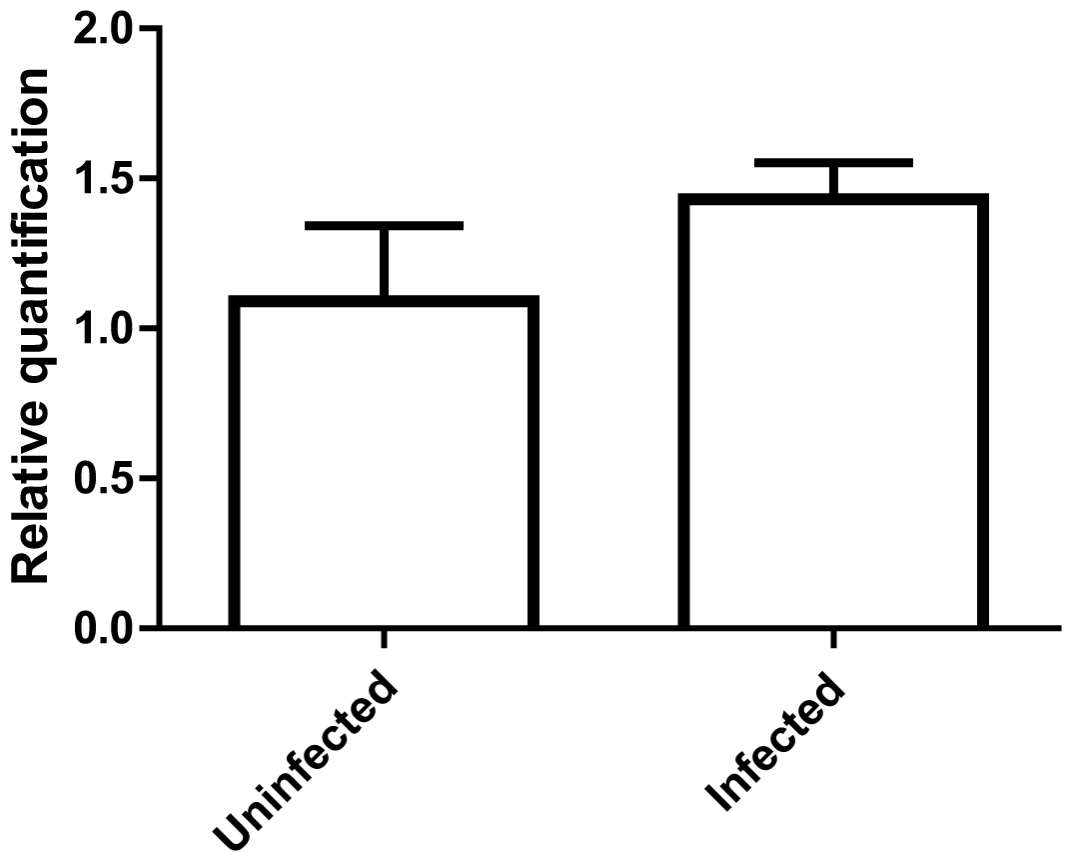
qRT-PCR of infestin. Adult insects infected with *T. cruzi* and uninfected *T. infestans* were used for analysis (three biological samples were used for both the uninfected and infected groups). All data were normalized to 18S ribosomal RNA, representing the mean (n = 3) of identical triplicates ± standard deviation. An unpaired *t* test was performed for statistical analysis.

Transcripts encoding infestin, an anticoagulant protein from the *T. infestans* midgut that counteracts the host's hemostastic system by inhibiting thrombin and factor XIIa from coagulation cascades [31], [32], were found in our analysis (Data S1, contig 256). Most of the activity of infestin-like inhibitors was found in the anterior midgut, suggesting that these anticoagulant molecules are synthesized and stored in the anterior midgut prior to the ingestion of blood [33]. No significant alterations in infestin expression were detected under *T. cruzi* infection (Figure 5). According to Lovato et al. [34], differences in infestin expression were observed 12 h after *T. cruzi* challenge, although the expression remained constant 36 h after infection. In our work, midguts were dissected 24 h post-challenge; therefore, we suggest that infestin expression can fluctuate at different times of infection. We cannot exclude the possibility that infestin may play a role in the first hours after *T. cruzi* infection, which could explain the presence of proteins prior to the blood meal.

Some transcripts had significant matches to different insect lipocalins. These proteins play several roles, including the transport of small molecules in vertebrates and invertebrates [35]. In our set of ESTs, a contig was found that is related to fatty acid binding lipocalins (FABL) (Data S1, contig 457), which may be related to the transport of fatty acids [36]. Moreover, the contig identified was related to triabin-like lipocalins, which are thrombin inhibitors [37]. Triabin-like lipocalins were identified in salivary glands transcriptomes from *T. infestans* and *T. dimidiata* [15]–[16].

Defensin ESTs were also detected in the present EST analysis (Data S1, contig 582). Defensins are antimicrobial peptides involved in defense against infection with microorganisms [38]. We observed a similar expression profile in libraries from both uninfected and *T. cruzi* infected triatomines. Defensin expression is upregulated in the posterior midgut of triatomine insects upon *T. cruzi* infection. However, this antimicrobial peptide is not modulated by *T. cruzi* in the stomach, and it may reflect a *T. infestans* adaptation to control symbiont multiplication [9].

Another putative protein, juvenile hormone binding protein, was identified. This protein is involved in the development and reproduction of insects and was proposed as a potential target to control the vector insect and consequently decrease the transmission of Chagas disease [11].

In summary, this work provides the first global analysis of expression profiles from the midgut of a Chagas disease vector under *T. cruzi* infection, with a resulting repertoire of transcripts that are important in the elucidation of metabolic processes in *T. infestans*. We demonstrated differential expression of several ESTs upon *T. cruzi* infection. Moreover, we reported a largely upregulated putative 14 kDa protein that has not been described previously. Together, the data provide relevant information regarding the interaction of *T. cruzi* with the vector insect and new target molecules for future research in the control of Chagas disease.

## Materials and Methods

### Ethics Statement

Experimental protocols for mouse infections were carried out in accordance with the guidelines of the Ethics Committee in Research from the Federal University of São Paulo (CEP – UNIFESP), approved under registry 1850/08.

### Insects and infection protocol

*T. infestans* were reared under controlled temperature (27±2.0°C) under a 12/12 light/dark cycle. Adult male insects (n = 10) that had been starved for 30 days were allowed to feed *ad libitum* on anesthetized mice (ketamine 150 mg/kg and xylazine 7 mg/kg). Insect tissues were dissected 24 h after feeding. For infection experiments, insects were infected orally by feeding *ad libitum* on anesthetized mice infected with *T. cruzi* Y strain, and these insects were also dissected 24 h after feeding. Mice infection was performed according to Kollien and Schaub [39], and the population density (1×10^6^ parasites/mL) was determined using a Neubauer chamber.

### cDNA library construction

*T. infestans* mRNA was extracted from 10 anterior midguts from insects belonging to the control group (uninfected insects) and 10 anterior midguts from the infected insects group using TRIZOL reagent (Invitrogen, Carlsbad, CA). The PCR-based cDNA library was created following the guidelines of the SMART cDNA library construction kit (Clontech), which provides an oligonucleotide named SMART IV in the first-strand synthesis to produce a high percentage of full-length, double-stranded cDNA. *T. infestans* midgut total RNA was used for reverse transcription to cDNA using MMLV reverse transcriptase (Clontech), the SMART IV oligonucleotide, and the CDS III/primer (Clontech). The reaction was carried out at 42°C for 1 h.

A long-distance PCR-based method was utilized to perform the second-strand synthesis by using Advantage Taq polymerase mix (Clontech), a 5′ PCR primer and a CDS III/3′ primer, which inserts *Sfi*1A and B restriction enzyme sites at the end of the cDNA. The PCR conditions were 95°C for 1 min, 19 cycles of 95°C for 15 s and 68°C for 6 min. A 5-µL sample was analyzed on a 1.1% agarose/EtBr (0.1 mg/mL) gel to check the quality and abundance of the cDNA. Next, DNA polymerase was inactivated with proteinase K, followed by precipitation, and double-stranded cDNA was then digested with *Sfi*I restriction enzyme at 50°C for 2 h. Then, cDNA was fractioned on a ChromaSpin-400 column (Clontech). The fractions were analyzed on a 1.1% agarose/EtBr (0.1 mg/mL) gel, and fractions containing cDNA were pooled. The cDNA was precipitated and ligated into a λTriplEx2 vector (Clontech), and the ligation was packaged using GigaPack Gold III Plus packaging extract (Stratagene) according to the manufacturer's guidelines. The packaged library was plated by infecting log-phase XL1-Blue *Escherichia coli* cells (Clontech) for cDNA library amplification and titering unamplified and amplified libraries.

### Sequencing of *T. infestans* cDNA libraries

The *E. coli* BM 25.8 strain was used to inoculate 2 mL of LB broth medium at 31°C with shaking at 180 rpm until the OD_600_ reached 1.3. Next, MgCl_2_ was added to the culture (10 mM final concentration). Then, the bacterial culture was combined with separate amplified cDNA libraries, and the mixture was incubated at 31°C without shaking. Next, 400 µL of LB medium and the bacteria, including the libraries, were incubated for an additional 1 h at 31°C with shaking (225 rpm). Finally, infected cells (1–10 µL) were spread on an LB/ampicillin plate and grown overnight at 31°C to obtain isolated clones, maintaining the excised pTriplEX2 containing the DNA inserts.

Following an excision protocol, mini plasmidial preparations (minipreps) were performed using bacteria BM 25.8 clones according to Sambrook [40]. Then, minipreps (200 ng) were used as templates for sequencing reactions. The primer used was upstream from the inserted cDNA (LD insert 5′-CTCGGGAAGCGCGCCATTGTGTTGGT-3′), and the sequencing reaction was performed on an MJ PT-200 thermocycler. The sequencing products were precipitated using ethanol and sodium acetate buffer. Finally, DNAs were sequenced on an ABI 3130 sequencer (Applied Biosystems).

### Bioinformatics analysis

Expressed sequence tags (ESTs) were trimmed of primer and vector sequences and then assembled and compared with other databases using programs from the National Center for Biotechnology Information (NCBI). The cleaned, non-assembled sequences were deposited in dbEST from NCBI under accession numbers JK733006 – JK733438 and JK733439 – JK733877 for ESTs from uninfected and infected *T. infestans* libraries, respectively.

The BLAST tool [41] and CAP3 assembler [42] were used to compare and assemble the sequences. For functional description of the transcripts, the Blast tool [43] was used with a program [44] developed by Jose Ribeiro (NIAID – NIH) to compare the sequences with the following databases: Conserved Domain Database (CDD) [45]; Protein families (Pfam) [46]; Ortologous eukaryotic domains (Kog) [47]; simple modular architecture tool (Smart) [48] using rpsBlast; Mit-pla and ribosomal RNA (rRNA) using BlastN and Swissprot; Gene Ontology (GO) [49]; and a subset of the Non-Redundant Database (NR) that we called NR-light using BlastX. This subset of the non-redundant database comprises proteins from the organisms having the following starts (or ends, when the bracket indicates closure): “[Tribolium”, “[Apis m”, “[Anopheles”, “[Aedes”, “[Culex”, “[Ixodes”, “[Glossina”, [“Ochlerotatus”, “[Tabanus”, “[Chrysops”, “[Amblyomma”, “[Ornithod”, “[Argas”, “[Rhipicephalus”, “[Boophilus”, “[Phlebotomus”, “[Lutzomyia”, “[Simulium”, “[Rhodnius”, “[Panstrongylus”, “[Triatoma”, “[Dipetalogaster”, “[Mus m”, “[Nasonia”, “[Strongylocentrotus”, “[Daphnia”, “[Homo sa”, “[Arabidopsis”, “[Escherichia”, “[Pseudomonas”, “[Streptococcus”, “[Acyrthosiphon”, “[Pediculus”, “[Ciona”, “[Danio”, “[Caenorhabditis el”, “[Drosophila mela”, “[Plasmodium”, “[Haemaphysalis”, “[Cimex”, “[Rickettsia”, “[Asaia”, “[Klebsiella”, “[Serratia”, “[Enterobacter”, “[Trypanosoma”, “[Leishmania”, “virus]”, “[Saccharomyces”, “[Neurospora”, “[Aplysia”, “[Babesia”, “[Toxoplasma”, “[Nocardia”, “[Rhodococcus”, “[Streptomyces”, “[Ceratitis”, “[Hyalomma”, “[Brugia”, “[Branchiostoma”, [Bos ta”, “[Gallus g”, “[Hydra”, “[Orizasa”, “[Nephila”, “[Titus”, “[Sussc”, “[Rattusra”, “[Canisfa”, “[Argiope”, “[Araneus”, “[Acanthoscurria”, “[Agelenopsis”, “[Schistosoma”, “[Bombyxmor”, “[Bothrops”, “[Ancylostoma”, “[Necator”, “[Bungarus”, “[Crotalus”, “[Hirudo”, “[Desmodus”, “[Xenopsylla,” “[Ctenocephalides” and “[Caenorhabditis elegans”.

The sequences were also blasted against a *Rhodnius prolixus* protein database (Data S1). This database was built through automatic gene previsions obtained using GeneID software [50] and trained with a protein dataset deduced from an extensive Rhodnius transcriptome (data not published but available at http://rhodnius.iq.ufrj.br/English/index.php?option=com_content&view=article&id=3&Itemid=4). GeneID training and protein prediction (data not published) were performed by Dr. Rafael Dias Mesquita (IQ-UFRJ-Brazil), who kindly allowed us to use this information. The ESTs from the two libraries, non-infected insects and insects infected with *T. cruzi*, were assembled together. However, we controlled the quantity of reads from each library that were assembled to form each cluster. This procedure enabled us to predict which clusters would have downregulated, upregulated or unchanged expression upon infection with *T. cruzi*.

Another program, kindly provided by Dr. José Marcos Ribeiro (NIAID – NIH), was used to organize the blast results. This program inserted the relevant information into a column-hyperlinked excel spreadsheet (Data S1). One of these programs, named “Classifier,” was used to read all blast results for each cluster; it was also used to functionally classify and then propose names for the proteins potentially coded by the cluster.

Another program, assembly joiner, extracted the coding sequences (CDSs) from the clusters, eliminated the 5′ and 3′ UTRs when present and corrected the frameshifts by substituting stop codons in the middle of each sequence. In addition, this program deleted the truncated codon by replacing the unknown triplet with X in the amino acid sequence. The resulting protein sequences were also blasted against the databases cited above, and the blast results were listed in another excel spreadsheet (Data S2). In this spreadsheet, there are five worksheets as follows: the first shows the most reliable data (<5% X), the second shows all results, and the other three show the separate analyses of the upregulated, downregulated and unchanged clusters.

### Quantitative RT-PCR (qRT-PCR)

Quantitative RT-PCR (qRT-PCR) was performed using three biological samples for each group (infected and uninfected insects), each obtained from a pool of four insects. Total RNA was extracted from the anterior midgut using TRIzol reagent (Invitrogen) and quantified using NanoVue equipment (GE Healthcare). Then, 1 µg of RNA was treated with 1 Unit of DNase (Fermentas) for 1 h at 37°C. Reactions were stopped by adding EDTA and heating for 10 min at 65°C. cDNA synthesis was performed using the ImProm-II™ Reverse Transcription System (Promega) following the manufacturer's guidelines.

Quantitative RT-PCR was performed following the methods described by Livak and Schimittgen [51] for delta delta Ct calculations to conduct relative quantification of the transcripts. The calibrator was the uninfected group for upregulated transcripts and the infected group for downregulated transcripts. Anterior midgut cDNAs from *T. cruzi*–infected and non-infected *T. infestans* were quantified using SYBR® Green PCR Master Mix (Applied Biosystems) in a 7500 Real-Time PCR System (Applied Biosystems). The qRT-PCR reaction consisted of 1 µL of 10-fold diluted cDNA (5 ng), 6 µL of SYBR® Green and 0.3 µM of each primer (Primer sequences are in Data S3) in a 12 µL total volume. 18S ribosomal RNA was used as the internal control. The PCR program comprised 40 cycles at 94°C (15 seconds) and 60°C (1 min), followed by melt curve generation. Melt curves were analyzed to check the specificity of amplification. Reactions were performed in triplicate (for each biological sample), and all values are represented as the mean ± standard deviation. An unpaired t test was conducted for statistical analysis, and a significant difference was accepted at P<0.05.

## Supporting Information

Data S1 **(TI-S1) Hyperlinked excel file with assembled contigs and spreadsheets containing information about the best hits in databases and a classification of contigs.**
(XLSX)

Data S2 **(TI-S2) Hyperlinked excel file with coding sequence information.**
(XLSX)

Data S3 **(qRT-PCR) Word file containing primer sequences for qRT-PCR expression analysis.**
(DOCX)
