# Supplementary material for: Differential Expression Profiles in the Midgut of Triatoma infestans Infected with Trypanosoma cruzi
Source: PLoS One. 2013 May 2;8(5):e61203. doi: 10.1371/journal.pone.0061203 (PMC3642171; doi:10.1371/journal.pone.0061203)
Supplement: Data S3 — (qRT-PCR) Word file containing primer sequences for qRT-PCR expression analysis. (DOCX) [file pone.0061203.s003.docx]

**Primer sequences:**

| Target | Sequence |
| --- | --- |
| 18S ribosomal forward | 5’-GCGCGGCGGGGGCATTCGTATTG-3 |
| 18S ribosomal reverse | 5’-ATCGCTGGCTGGCATCGTTTATTTGCGG-3’ |
| 15 kDa protein forward | 5'-GGGAATTCCATATGTATCCAAAAACTGCATGC-3' |
| 15 kDa protein reverse | 5'-AACATTTATGGAAGATAAGGATCCGCG-3' |
| Nitrophorin-like forward | 5'-CCGCTCGAGTTATTTCAAAATTGTTTC-3 |
| Nitrophorin-like reverse | 5'-CGCATTTTTTGGAATGTAAGTTGTACCTAG-3' |
| Cathepsin D forward | 5'-GGAACTTTTGTCTCCAACAATGGAAAC-3' |
| Cathepsin D reverse | 5'-TAATGATATTTCACCATAGTATGCGGAGTT-3 |
| Lysozyme forward | 5'-TTTCTGGGCGTTTCAGAGGCGAGAGTT-3' |
| Lysozyme reverse | 5'-ATGACAATCATGTCCAGGTTTACCATACGT-3' |
| Thioredoxin reductase forward | 5'-TTTTTCATACCTCAGCGTAATCCTGCA-3' |
| Thioredoxin reductase reverse | 5'-CGTTGGATGAATGCCTACTGTATTTTC-3' |
| Infestin forward | 5'-GATCCTTGTGAATGCCCTCGAGCTCTA-3' |
| Infestin reverse | 5'-CGGTGTAGAACTCGTGGGCATGCACA-3' |
